# Automatic Evaluation of Soybean Seed Traits Using RGB Image Data and a Python Algorithm

**DOI:** 10.3390/plants12173078

**Published:** 2023-08-28

**Authors:** Amit Ghimire, Seong-Hoon Kim, Areum Cho, Naeun Jang, Seonhwa Ahn, Mohammad Shafiqul Islam, Sheikh Mansoor, Yong Suk Chung, Yoonha Kim

**Affiliations:** 1 Department of Applied Biosciences, Kyungpook National University, Daegu 41566, Republic of Korea; ghimireamit2009@gmail.com (A.G.); shafik.hort@gmail.com (M.S.I.); 2 National Agrobiodiversity Center, National Institute of Agricultural Sciences, RDA, Jeonju 5487, Republic of Korea; shkim0819@korea.kr; 3 School of Applied Biosciences, Kyungpook National University, Daegu 41566, Republic of Korea; nina0821@naver.com (A.C.); nangni99@naver.com (N.J.); ash8235@naver.com (S.A.); 4 Department of Plant Resources and Environment, Jeju National University, Jeju 63243, Republic of Korea; mansoorshafi@jejunu.ac.kr; 5 Upland Field Machinery Research Center, Kyungpook National University, Daegu 41566, Republic of Korea

**Keywords:** image analysis, Python algorithm, soybean, seed number, seed size

## Abstract

Soybean (*Glycine max*) is a crucial legume crop known for its nutritional value, as its seeds provide large amounts of plant protein and oil. To ensure maximum productivity in soybean farming, it is essential to carefully choose high-quality seeds that possess desirable characteristics, such as the appropriate size, shape, color, and absence of any damage. By studying the relationship between seed shape and other traits, we can effectively identify different genotypes and improve breeding strategies to develop high-yielding soybean seeds. This study focused on the analysis of seed traits using a Python algorithm. The seed length, width, projected area, and aspect ratio were measured, and the total number of seeds was calculated. The OpenCV library along with the contour detection function were used to measure the seed traits. The seed traits obtained through the algorithm were compared with the values obtained manually and from two software applications (SmartGrain and WinDIAS). The algorithm-derived measurements for the seed length, width, and projected area showed a strong correlation with the measurements obtained using various methods, with R-square values greater than 0.95 (*p* < 0.0001). Similarly, the error metrics, including the residual standard error, root mean square error, and mean absolute error, were all below 0.5% when comparing the seed length, width, and aspect ratio across different measurement methods. For the projected area, the error was less than 4% when compared with different measurement methods. Furthermore, the algorithm used to count the number of seeds present in the acquired images was highly accurate, and only a few errors were observed. This was a preliminary study that investigated only some morphological traits, and further research is needed to explore more seed attributes.

## 1. Introduction

Soybean (*Glycine max* L. Merr.) is the main legume crop primarily consumed as a major plant protein source by both humans and livestock [1,2]. To obtain higher soybean yields, good quality seeds are a major prerequisite. Thus, it is essential to select first-grade soybean cultivars based on the seed size, shape, color, and absence of physical or pathogen-derived damage [3]. The seeds’ morphological traits and their correlation are important tools for genotype discrimination and for breeding high-yielding seeds [4]. Seed size is not only an important parameter for the identification of quantitative trait loci, detection of biotic and abiotic stress, and hormonal control, but it also significantly affects the growth of seedlings in relation to the external environment [5,6]. The morphological and physiological properties of seeds play a vital role in germination and plant growth [7]. Apart from seed size, seed number is another important agronomic parameter used for estimating the grain weight and yield and has an essential role in soybean breeding [8]. The total number of seeds per plant, total seeds per selected area, and seeds per pod are the main traits used for estimating the yield of soybean [9].

With the recent development of different image processing tools, various image-based studies have been conducted to measure soybean traits. For example, digital RGB images have been used to estimate the grain yield, leaf biomass, and canopy cover [10]. Lay et al. reported a method to detect disease in soybean using spectral images [11]. Similarly, another study used digital image-based machine learning to detect root nodules in soybean [12]. Several studies have used image-based high-throughput phenotyping technology to estimate the morphological traits of soybean seeds, including the shape, length, width, area, perimeter, and seed coat color [13]. Baek et al. [14] tested the seed viability using hyperspectral images. In another study, the image-based subscription software GrainScan was used to determine the seed size and color in wheat and *Brachypodium distachyon* [15]. The WinSEEDLE system (Reagent Instruments Inc., Quebec, QC, Canada) also measures a wide range of seed parameters based on image analysis, but it comes at a high cost. Tanabata et al. developed a C++ language-based program known as SmartGrain for the analysis of seed shape using images [16]. Quantitative-plant.org, a popular website containing information related to various plant trait analysis software, lists only eight programs for the analysis of seed traits, three of which require subscription [17]. As a result, very few studies have analyzed seed traits, although they are among the most important morphological parameters in plants.

Soybean seeds are small in size and irregular in shape, which makes their manual measurement particularly tedious. The major seed traits usually measured are the seed length and width, and the easiest way to measure them is by using calipers. However, this procedure is time consuming and difficult due to the large volume of data [16]. Similarly, the manual counting of seeds is also a laborious task prone to human error, and counting devices are costly for regular researchers. The recent advancements in image processing and machine learning can overcome these difficulties and greatly facilitate seed trait measurement [18]. For the automatic analysis of seed morphological traits, the present study aimed to analyze digital images of soybean seeds to measure major morphological traits, i.e., seed length, seed width, seed projected area (PA), and aspect ratio (or eccentricity index), and to count the total number of seeds present in each acquired image using a simple Python algorithm without incurring in any recurring expenses.

## 2. Results

### 2.1. General Distribution and Fit of Plot

**Figure 1:**
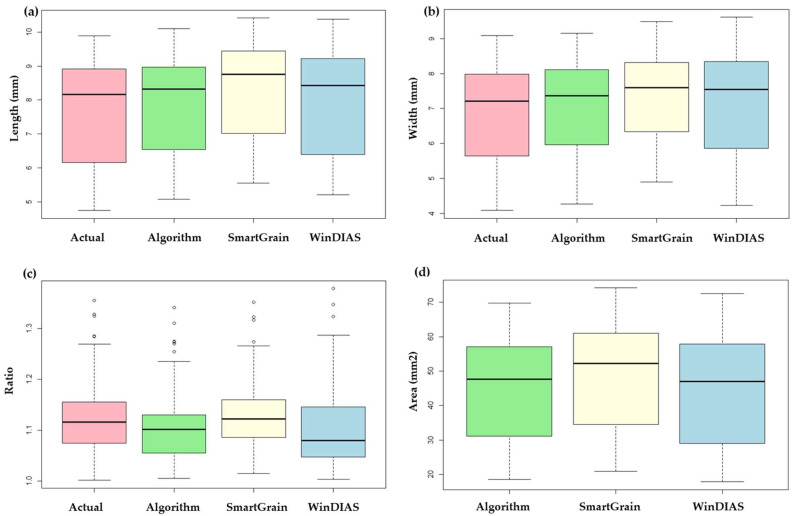
Comparison of boxplots for data distribution between the actual, algorithm, SmartGrain- and WinDIAS-derived measurements: (**a**) seed length, (**b**) seed width, (**c**) aspect ratio, and (**d**) PA.

Data distributions were generally compared using boxplots, as shown in Figure 1. The algorithm-generated values (seed length, width, aspect ratio, and PA) were compared with the actual (manually measured), WinDIAS 5.3 (Delta-T Devices, Cambridge, UK), and SmartGrain [16]-generated values. The box plot for the length (Figure 1a) shows that the actual, algorithm, and SmartGrain-generated values have almost a similar size indicating a similar distribution of the data among them. While a slight difference in box size was observed in the WinDIAS-generated length. A similar result was observed in the box plot of the width (Figure 1b), where the median line (dark black colored line between the boxes) also had almost same position. Although, the box size of the ratio (Figure 1c) was similar in size, the median lines were in different positions along with some outliers indicating a difference in distribution of the data among the methods. The distribution of data in the PA was similar, as shown in Figure 1d.

**Figure 2:**
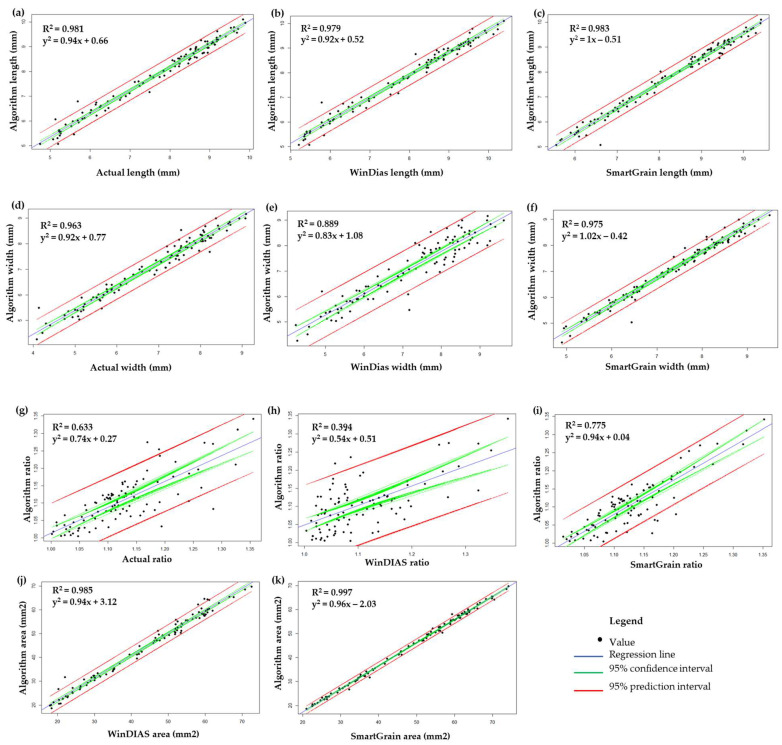
Fit of plot for the algorithm-derived values against the actual, WinDIAS-, and SmartGrain-derived values. (**a**) Actual length, (**b**) WinDIAS length, (**c**) SmartGrain length, (**d**) actual width, (**e**) WinDIAS width, (**f**) SmartGrain width, (**g**) actual ratio, (**h**) WinDIAS ratio, (**i**) SmartGrain ratio, (**j**) WinDIAS area, and (**k**) SmartGrain area.

To better determine the accuracy of the algorithm-derived values, we created a fit of the plot within a 95% confidence interval and a 95% prediction interval against the actual, SmartGrain- and WinDIAS-derived values (Figure 2). The algorithm-generated value was highly correlated with the SmartGrain-generated values. The R^2^ was highest among all the other methods, i.e., 0.983, 0.975, 0.775, and 0.997 (*p* < 0.0001) for the length (Figure 2c), width (Figure 2f), aspect ratio (Figure 2i), and PA (Figure 2k), respectively. Here, the values seemed to be predominantly concentrated within the 95% confidence interval with very few outliers outside 95% prediction interval. Similarly, a high correlation was observed for the actual (Figure 2a,d) and WinDIAS-(Figure 2b,e,j) derived value, where the R^2^ was greater than 0.95 in all the seed parameters except for the aspect ratio (Figure 2g,h). The slope of the line was around 1 with a lower intercept value in all cases except for the aspect ratio determined by WinDIAS (Figure 2h), indicating a good conformity between the algorithm-derived values and the other derived values.

### 2.2. Error Calculation

Different error types, i.e., the residual standard error (RSE), the root mean square error (RMSE), and the mean absolute error (MAE), were calculated to evaluate the accuracy of the measurements (Table 1). All the methods exhibited error rates of less than 0.5% for the seed length, width, and aspect ratio measurements. Among these methods, SmartGrain yielded the lowest RSE at 0.189% for the seed length, while the WinDIAS measurement had the smallest RMSE at 0.249% and MAE at 0.194% for the same parameter. When assessing seed width, SmartGrain exhibited the smallest error, and for the aspect ratio, the smallest error was observed compared with the actual measurements. Similarly, for the PA, error rates of less than 4% were observed when compared to the SmartGrain and WinDIAS measurements. Although the R^2^ value for the aspect ratio was minimal compared with those for the other traits, the error values (RSE, RMSE, and MAE) were also the lowest. This might be due to the fact that the range of data for the algorithm-derived ratio was between 1.0 and 1.34, while that for the ratio obtained from other measurements was between 1.0 and 1.37. As these error values depend on the mean, having a very small range of data would generate a lower error.

### 2.3. Seed Number

**Figure 3:**
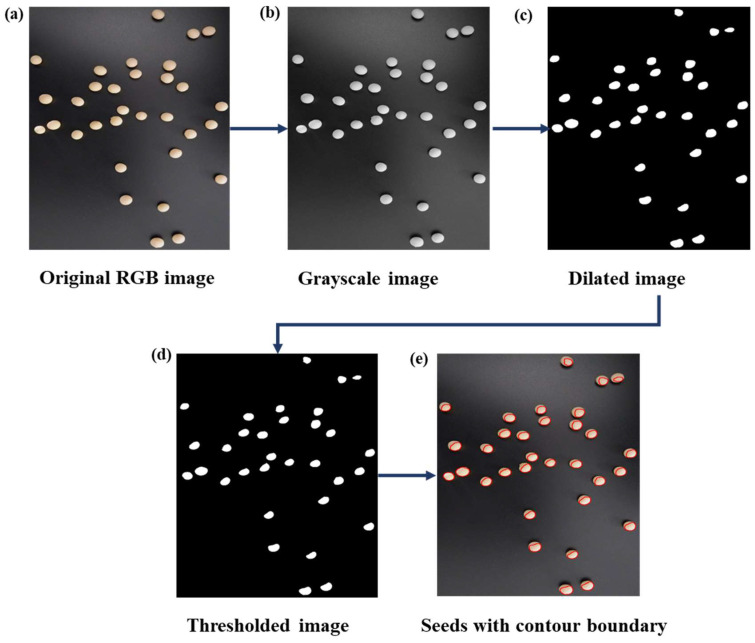
Different steps followed to count the total number of seeds present in an image: (**a**) original RGB image, (**b**) grayscale image, (**c**) dilated image, (**d**) thresholded image, and (**e**) image showing seeds with contour boundaries.

The progressive steps taken to count seed numbers are shown in Figure 3. The images were first preprocessed by converting the RGB images (Figure 3a) into grayscale images (Figure 3b); then, the grayscale images were dilated (Figure 3c) and thresholded (Figure 3d). Contour boundaries were created on the seeds in the thresholded images (Figure 3e); then, by counting the total number of contours in each image, the total number of seeds per image was obtained. The total seed numbers derived from manual counting and from the algorithm are reported in Table S1. While taking the photographs, it was essential to remove any background noise, as this could be misinterpreted as seeds during analysis. Similarly, the overlapping of seeds during image acquisition could also generate error as the overlapped seeds would be considered as a single seed (Figure S1).

## 3. Discussion

The size and shape of seeds are key factors in agronomy, as they affect the eating quality, yield, and market price. Accurate assessment of these morphological traits, both externally and internally, is important for advancements in plant research areas such as genetics, physiology, functional analysis, and plant breeding [16]. The seed lengths, widths, aspect ratio, and PA obtained from the algorithm were almost similar to the manual, SmartGrain, and WinDIAS measurements showing a good conformity between the algorithm-derived and differently derived values. The fit of the plot, the high R^2^ values, the low error percentages, and the ease of analysis suggest that this Python-based image analysis of seed traits performs better than the manual method. An android application platform based upon the OpenCV library was developed by Wu et al. [19], which evaluated the thousand-kernel weight with an error percentage of less than 3%. Soybean seed morphological trait evaluation along with the prediction of the hundred-seed weight can also be conducted using a machine learning algorithm [20]. A similar study based on a convolution neural network (CNN) for the evaluation of phenotypic traits of soybean seed along with the identification of damaged and diseased seeds was conducted by Song et al. [21]. The application of CNN was also used to classify the normal, damaged, and abnormal soybean seeds with an accuracy of more than 95% in all instances [22]. A previous study of lentils reported similar high R^2^ values of >0.95 for the seed size measured both manually and using an image analysis algorithm [23]. Shahin et al. [24] also reported a high measurement accuracy while measuring the seed size and shape of lentils, with R^2^ values of approximately 0.90 and an RMSE of <2%. Likewise, the image-based measurement of chickpea seed size revealed a correlation coefficient of 0.90 when the image analysis method was compared to the ground-truth data [25]. A deep learning method for the estimation of seed size in rice yielded an MSE lower than 0.11 compared with different imaging programs (ImageJ, GrainScan, and GridFree) [26]. GrainScan an image-based seed trait analysis program had an average accuracy of 0.993 for the seed area, 0.981 for the length, and 0.990 for the width [15]. Similarly, seed counting based on digital images also showed high accuracy in the current study, where some errors were observed due to noise and overlapping seeds. In the case of rice grain phenotyping, Duan et al. created a labor-free machine that integrated spikelet threshing, grain imaging, and real-time algorithm-based evaluation of grain traits. This system allowed measuring grain traits, such as length, width, 1000-grain weight, and seed packing for each rice plant, without the need for manual intervention, and produced an MAE of less than 5% [27]. A similar, but slightly less accurate result was obtained for seed counting when the seeds overlapped each other [28].

As the code runs in the Python environment, there is no requirement of additional hardware or software for the analysis, and the analysis can be performed in any operating system, for example, Windows, macOS, and LINUX. Programs like GrainScan, Lemna Launcher, SeedCount, SmartGrain, and WinSEEDLE are supported in Windows operating systems only. According to Lobet [29], many studies have been conducted in plant image analysis techniques; however, 25% of them were not validated, only 31% of them were accessible, and only 39% of the tools were still maintained. Thus, there is a need for research that does not require regular maintenance, is easily accessible, and is validated. This study tries to incorporate all the above mentioned parameters. Another feature of this set of algorithms is that no manual annotation of the seed image is needed. Automatic thresholding of the image is performed as result of which manual annotation is not required. This kind of annotation is required in software like WinDIAS. Similarly, a manual change in the threshold value can also be made, if the proper contour boundary is not obtained while analyzing the seed image. Likewise, any digital images can be analyzed using these set of algorithms provided that the calibration value is set without any additional hardware or accessories requirement. Software like WinSEEDLE require accessories like scanners and trays for the analysis of the seed traits (https://regentinstruments.com/assets/images_winseedle/WinSEEDLE_Brochure.pdf, accessed on 11 August 2023). Programs like SeedCount, a digital imaging system, require a flatbed scanner for analyzing the seed traits, and they are specially designed for the grain industry [30]. CNN and transfer learning-based phenotyping of seeds, although giving more phenotypic traits in a large scale, still have a high computational cost and time [31]. Although the study uses well known modules of OpenCV, we incorporated these into a single platform for the convenient and cost-free analysis of seed traits so that any research limited due to cost, additional accessories, and computing knowledge could be carried out easily.

Digital image-based seed counting is a convenient and time-saving approach, but further research is required to improve its accuracy, as it is still in the development phase. Currently, seed counting using RGB image analysis is the primary method employed. However, incorporating deep learning or machine learning techniques in combination with image processing can serve as an alternative approach to enhance accuracy. Presently, only four major seed traits are measured, and the total seed numbers are counted. Further research is needed to accurately quantify more seed traits.

## 4. Materials and Methods

### 4.1. Image Acquisition and Thresholding

A total of 20 different soybean cultivars were used for image acquisition and subsequent measurement of the morphological traits of seeds (Table S2). These cultivars were selected based on different seed sizes. Specifically, sizes ranging from 4 to 9 mm in width and length were selected to minimize biases. The images were acquired using a digital camera (Canon, EOS 200 M200, Tokyo, Japan) on a black background. The camera settings were ISO speed, ISO 1600; F-stop, f/6.3; focal length, 45 mm; and image size, 6000 × 4000. Five seeds per cultivar were photographed, for a total of 100 images. To count the number of seeds, these were randomly placed on a smooth plain black surface that presented no background noise.

**Figure 4:**
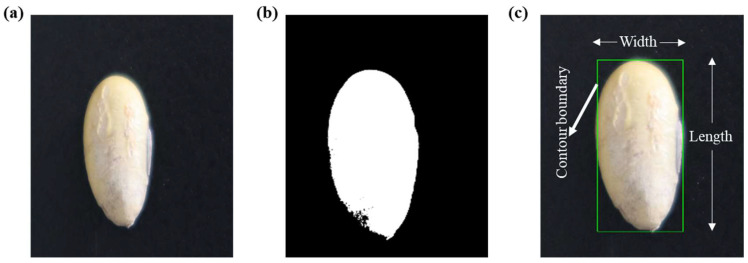
Image processing during data extraction for the measurement of seed attributes: (**a**) original RGB image, (**b**) thresholded image, and (**c**) contoured image with contour boundary.

The thresholding of an image refers to the segmentation of the desired image part from the background. In other words, it is a method to differentiate the desired portion from the background using a certain threshold value. In this study, each RGB image (Figure 4a) was thresholded to differentiate the seed from the background (Figure 4b). The global threshold of 140,255 was used for the seed length and width evaluation, and 170,255 was used for PA estimation and seed counting. The high threshold for the PA evaluation and seed counting was set to avoid any background noise, which can be interpreted as seeds while counting total number of seeds, hence causing errors.

More than just one seed can be used to analyze the seed traits by specifying the number of seeds in the given code; the rest of the steps are the same as those for the analysis of a single seed. The detail code is provided in the Supplementary File.

### 4.2. Programing Language

The image analysis algorithm was written using Python 3.9.12 as a programming language and Spyder 5.2.2 as the working environment. The contour detection function of the OpenCV module was used for extracting the contour boundaries of the seed images (Supplementary File). After the image was thresholded, a rectangle contour was drawn around the seed. Only the contour line corresponding to the maximum area was extracted; so, the contours made on the noises were automatically eliminated (Figure 4c). For the PA, the countNonZero function was used to count the total number of pixels of the thresholded seed image. Similarly, the same contour detection function was used to count the seeds through the creation of contour boundaries around them, providing the total number of seeds present in each image (Supplementary File).

The lengths and widths of seeds obtained using the contour detection method and PA obtained from the countNonZero function were only expressed as pixel numbers. A separate algorithm was used to convert these numbers into standard measurements. Specifically, the MousecallBack function of OpenCV was used to calibrate the number of pixels present on a definite point. This function calculates the total number of pixels present between two known distances based on the distance between coordinates. Keeping the same camera position and settings, we photographed a ruler and used the function to calibrate the number of pixels present within 10 mm. Finally, by dividing the total number of pixels calculated using the contour detection method (length and width) by the number of pixels present within 1 mm, we obtained the standard length and width measurements in mm, and dividing the number of pixels per mm^2^, we obtained the PA in form of mm^2^.

### 4.3. Validation and Statistical Analysis

For the cross validation, the algorithm-generated seed trait values were compared with actual values (manually measured values by Bluetech digital calipers, China) and two standard software WinDIAS 5.3 and SmartGrain. Since, the PA cannot be measured manually, it was compared with only the two-standard software. Similarly, for the validation of the seed numbers, the seeds were first counted manually and then were randomly placed on a black surface and photographed, and the values obtained using the two methods were compared. To compare the algorithm-derived values with the manual and software-derived values, we used the following statistical parameters: R^2^, RMSE, RSE, and MAE. The statistical analysis (box plot and fit of plot) was carried out using RStudio 2023.03.0 Build 386. The RMSE, MAE, and RSE collectively measure the effectiveness of a linear model in predicting the observed variable. Their respective formulas are:

(1) RMSE=1n∑i=1noi−si^2,

where *n* is the total number of observations, oi is the observed value for the *i*th observation, and si is the standard value for the *i*th observation;

(2) $$\mathrm{MAE}=\frac{1}{n}\sum_{i=1}^{n}\left|oi-si\right|,$$

where *n* is the total number of observations, oi is the observed value for the *i*th observation, and si is the standard value for the *i*th observation;

(3) RSE=∑i=1noi−si^2df,

where *n* is the total number of observations, oi is the observed value for the *i*th observation, si is the standard value for the *i*th observation, and *df* represents the degree of freedom.

## 5. Conclusions

The morphological traits of seeds, such as the length, width, PA, aspect ratio, and seed number, are essential from both the breeding and agronomic points of view. However, while these plant traits are important, their manual measurement is tedious and time consuming. Thus, we developed a user friendly and convenient image-based Python algorithm for seed trait measurement. The results showed that this simple algorithm, along with the calibration method, can be effective for seed phenotyping. In addition to quantifying the morphological traits (i.e., the length, width, PA, and aspect ratio) with a high accuracy, this method allowed the counting of the total number of seeds present in each acquired image.

## Figures and Tables

**Table 1:** Error values of different seed traits.

Parameters	Actual			SmartGrain			WinDIAS		
	RSE%	RMSE%	MAE%	RSE%	RMSE%	MAE%	RSE%	RMSE%	MAE%
Length	0.198	0.295	0.229	0.189	0.484	0.446	0.209	0.249	0.194
Width	0.243	0.366	0.292	0.200	0.326	0.282	0.418	0.484	0.362
Aspect ratio	0.042	0.049	0.034	0.330	0.039	0.028	0.054	0.066	0.050
PA	----	----	----	0.799	3.849	3.730	1.815	2.080	1.526

## Data Availability

Not Applicable.

## Supplementary Materials

The following supporting information can be downloaded at: https://www.mdpi.com/article/10.3390/plants12173078/s1, Figure S1: Errors that may arise due to the overlapping of seeds and noise; Table S1: Number of seeds counted manually and through the algorithm; Table S2: List of cultivars used for seed image acquisition; Python source code file.
