# Supplementary material for: Automatic Evaluation of Soybean Seed Traits Using RGB Image Data and a Python Algorithm
Source: Plants (Basel). 2023 Aug 28;12(17):3078. doi: 10.3390/plants12173078 (PMC10490075; doi:10.3390/plants12173078)
Supplement: Supplementary file 1 [file plants-12-03078-s001.zip › plants-2526309-supplementary.pdf]

Supplementary Materials:

Table S1: Number of seeds counted manually and using the algorithm.

Manual count	Algorithm count	Source of error
25	25	None
50	50	None
100	99	Overlapped seeds
150	150	None
250	250	None
300	299	Overlapped seeds
350	351	Noise counted as seed
400	400	None
450	451	Noise counted as seed
500	500	None

Table S2: List of cultivars used for seed image acquisition.

1. Singangkong	6. Uramkong	11. Zee	16. Zhonghuang 13
2. Daechankong	7. Taekwangkong	12.Sohwangkong	17. Rhosa
3. Jamolkong	8. Sinhwakong	13.Danmikong	18. Liao dou 13
4. Soyeonkong	9. Seunphungkong	14. Lang xiaoli douz	19. CS01093
5. Danmikong 2	10. Misokong	15. Jiyu 50	20. Jiyu 65

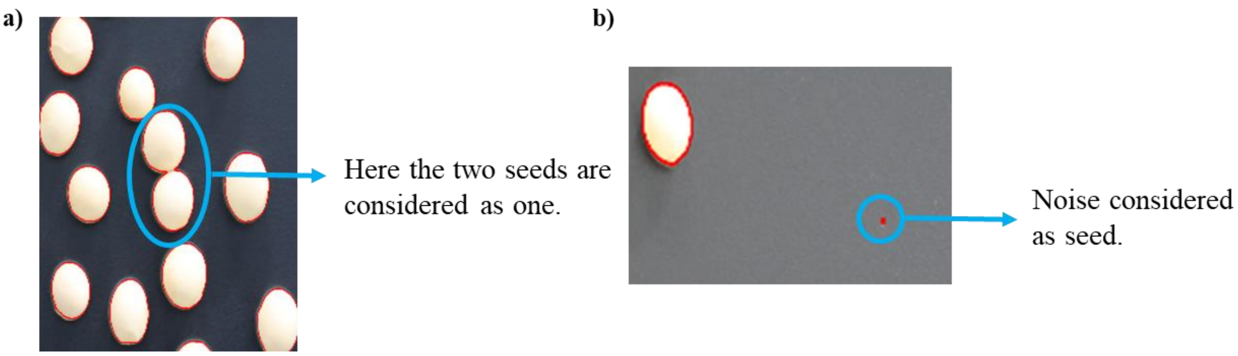

Figure S1. Errors that may arise due to overlapping seeds (a) and background noise (b).

Python Source Code.

a) Source code for seed size measurement single seed

```
import cv2 #import cv2 library

img = cv2.imread('D:/seed.jpg') #set the directory of the image

gray = cv2.cvtColor(img, cv2.COLOR_BGR2GRAY) #convert image to grayscale
ret, thresh = cv2.threshold(gray, 140, 255, cv2.THRESH_BINARY) #thresholding of the image, cv2.THRESH_BINARY_INV can be used if other image is taken in white background with different color seeds

contours, hierarchy = cv2.findContours(thresh, cv2.RETR_EXTERNAL, cv2.CHAIN_APPROX_SIMPLE) #find contours in thresholded image

max_area = 0

max_contour = None

for contour in contours: #selection of maximum contour length and width

    x, y, w, h = cv2.boundingRect(contour)

    area = w * h

    if area > max_area:

        max_area = area

        max_contour = contour

if max_contour is not None:

    x, y, w, h = cv2.boundingRect(max_contour)

    print('The width is:', w/69.1) #calculate width. The value 69.1 is from calibration.

    print('The height is:', h/69.1) # calculate length. The value 69.1 is from calibration.

    cv2.rectangle(img, (x, y), (x+w, y+h), (0, 255, 0), 2) #displayed contoured image with resizing

down_width = 1000

down_height = 1000

down_points = (down_width, down_height)

resized_down = cv2.resize(img, down_points, interpolation= cv2.INTER_LINEAR)

cv2.imshow('Resized Down by defining height and width', resized_down)

cv2.waitKey(0)
```

b) Seed size measurement for more than one seeds

```
import cv2 #import cv2 library
img = cv2.imread('D:/seed.jpg') #set image directory
gray = cv2.cvtColor(img, cv2.COLOR_BGR2GRAY) #convert image to grayscale
ret, thresh = cv2.threshold(gray, 170, 255, cv2.THRESH_BINARY) #use global threshold to thresh the image, cv2.THRESH_BINARY_INV can be used if other image is taken in white background with different color seeds
contours, hierarchy = cv2.findContours(thresh, cv2.RETR_EXTERNAL, cv2.CHAIN_APPROX_SIMPLE) #find the contours
max_areas = []
max_contours = []
for contour in contours: #find number of contours
    x, y, w, h = cv2.boundingRect(contour)
    area = w * h
    if len(max_areas) < 5: #set the number of seeds, here 5 seeds are used
        max_areas.append(area)
        max_contours.append(contour)
    else:
        min_area = min(range(len(max_areas)), key=max_areas.__getitem__) #get largest 5 contours
        if area > max_areas[min_area]:
            max_areas[min_area] = area
            max_contours[min_area] = contour
for contour in max_contours: #prints length and width of seeds serially
    x, y, w, h = cv2.boundingRect(contour)
    print("Width:", w)
    print("Height:", h)
    cv2.rectangle(img, (x, y), (x+w, y+h), (0, 255, 0), 2)
down_width = 1000
down_height = 1000
down_points = (down_width, down_height) #downsize the image and prints along with contour boundary
resized_down = cv2.resize(img, down_points, interpolation= cv2.INTER_LINEAR)
cv2.imshow('Contoured Image', resized_down)
cv2.waitKey(0)
cv2.waitKey(0)
```

c) Source code for seed number counting

```
import cv2 #import cv2

image = cv2.imread("D:/seed.jpg") #set image directory

gray = cv2.cvtColor(image, cv2.COLOR_BGR2GRAY) #convert RGB to grayscale

blurred = cv2.GaussianBlur(gray, (5, 5), 0) #use Gaussian blurr

ret, thresh = cv2.threshold(blurred, 170, 255, cv2.THRESH_BINARY) #global thresholding of image using 170,255 value

kernel = cv2.getStructuringElement(cv2.MORPH_RECT, (3, 3))

dilate = cv2.dilate(thresh, kernel, iterations=1) #dialtion
```

contours, _ = cv2.findContours(dilate, cv2.RETR_EXTERNAL, cv2.CHAIN_APPROX_SIMPLE) #**find the contours of dilated image**

img = image.copy()

cv2.drawContours(img, contours, -1, (0, 0, 255), 2) #**draw the contours**

number_seeds = len(contours) #**calculate contour number**

print ('The number of seeds present in the image is:' , number_seeds) #**prints number of seeds**

down_width = 1000

down_height = 1000

down_points = (down_width, down_height)

resized_down = cv2.resize(img, down_points, interpolation= cv2.INTER_LINEAR)

cv2.imshow('Contoured image', resized_down) #**displays contoured image**

cv2.waitKey(0)

cv2.waitKey(0)

d) Source code for seed projected area (PA)

import cv2 #**import library**

img = cv2.imread('D:/seed.jpg') #**set image directory**

gray = cv2.cvtColor(img,cv2.COLOR_BGR2GRAY)

ret, thresh = cv2.threshold(gray,170,255,cv2.THRESH_BINARY) #**thresholding of the image**

area = cv2.countNonZero(thresh) # **counting the total number of pixels of the thresholded image**

down_width = 1000

down_height = 1000

down_points = (down_width, down_height)

resized_down = cv2.resize(thresh, down_points, interpolation= cv2.INTER_LINEAR)

cv2.imshow('Thresholded image', resized_down) # **display the thresholded image**

cv2.waitKey(0)

cv2.waitKey(0)

total = area/(281*281) # **dividing the total pixels by number of pixels per cm^2. Here 281 is obtained from calibration.**

average = (total*100)/5 # **taking average of the 5 seeds for average projected area**

print('Average area of seeds is:', average) # **prints the average projected area of the seed**

e) Source code for calibration

```
import cv2 #import the library

import math

points = []

def mouse_callback(event, x, y, flags, param): #use of mouseCallback function

  global points

  if event == cv2.EVENT_LBUTTONDOWN: #click left button of mouse indicating the known points

    points.append((x, y))

    if len(points) == 2:

      print("Point 1 coordinates: ", points[0]) #displays first point coordinates

      print("Point 2 coordinates: ", points[1]) #displays second point coordinates

      dist = math.sqrt((points[1][0] - points[0][0]) ** 2 + (points[1][1] - points[0][1]) ** 2) #calculates the distance between the two points

      print("Number of pixels in between two points is: ", dist) #prints the number of pixels in that known points

img = cv2.imread("D:/scale.JPG") #set the directory of the image

cv2.namedWindow("image")

cv2.setMouseCallback("image", mouse_callback)

cv2.imshow("image", img)

cv2.waitKey(0)

cv2.destroyAllWindows()
```

***Note**- The sentence written after (#) sign in bold letters describes the function of the particular line of code.

Additional information on different metrics or values used in the line of code:

As for the seed trait measurement, the highest contour that represented only seeds was extracted so that a bit less threshold (140,255) value would also be able to segment the seeds from the background. The high threshold for area evaluation and seed counting was set to avoid any background noise, which can add the background noise as seed area and can be interpreted as seeds while counting total number of seeds, hence causing errors. If the contour boundary does not cover the seeds these threshold value can be changed according to the requirement. Similarly, the inverted binary thresholded can be done for different color seeds for the images taken in white background.

Likewise, the kernel size (K-size) value in Gaussian blur is a positive odd integer. The value (5,5), is commonly used for reasonable blurring for omitting the noise. The value (3,3) would provide less blurring effect, and a higher value of (9,9) or (11,11) would be stronger

The value of down size of image was chosen 1000*1000, which will facilitate the clear viewing of the picture during the analysis process. As the picture size was quite large (6000*4000), which does not get plotted in the python environment hence it was downsized to 1000*1000. User can set their own down size value.
